# Unintentional fatal toxicity due to nicotine chewing gum: A case report

**DOI:** 10.1097/MD.0000000000031225

**Published:** 2022-10-28

**Authors:** Jung Eun Lee, Tae Chang Jang, Young Woo Seo

**Affiliations:** a Department of Emergency Medicine, School of Medicine, Daegu Catholic University, Daegu, Republic of Korea.

**Keywords:** nicotine chewing gum, nicotine intoxication, respiratory failure

## Abstract

Since tobacco contains numerous harmful substances, besides nicotine, which is addictive, smoking cessation products and tobacco alternatives, such as electronic (e-) cigarettes, nicotine chewing gums, and patches, are being widely used. Nicotine gums are consumed orally. The nicotine from the gum is absorbed at a slower rate than that from e-cigarettes, and the former remains in the bloodstream for a longer period. In addition, the maximum number of daily doses is high, and it can be purchased without a doctor’s prescription in many countries.

**Patient concerns:**

A 29-year-old male patient consumed 5 2-mg nicotine gums at a time, twice a day, for 4 days (total amount: 70 mg). However, he visited the emergency unit with the chief complaint of involuntary limb movements after consuming an additional 15 gums 3 hour before the visit. At admission, his consciousness was clear, although 2 hour later, he experienced sudden loss of consciousness with worsening hypoxia and respiratory acidosis.

**Diagnosis:**

The patient’s vital signs were stable at the time of admission, and blood test results showed no specific findings other than a white blood cell count of 14,800/µL, lactate level of 6.4 mmol/L, and prolactin level of 119.02 ng/mL. In addition, chest radiography and head computed tomography scans showed no acute phase abnormalities. Two hours later, he experienced loss of consciousness and respiratory failure, and the results of blood tests performed at this time showed that his blood cotinine level was 3491 ng/mL.

**Interventions:**

Supportive treatment, including endotracheal intubation followed by mechanical ventilation, was provided.

**Outcome:**

The patient’s vital signs stabilized 3 days after treatment, and his consciousness and respiratory status had improved; therefore, mechanical ventilation was stopped. His condition was stable for the next 2 days, and he was discharged on the fifth day.

**Lessons:**

Acute respiratory exacerbation due to nicotine poisoning (from levels exceeding the lethal dose of 30–60 mg) was observed, although the gums were consumed over several days. Patients with nicotine poisoning may show acute respiratory failure and should be monitored carefully. Further studies are required to determine the toxic effects of nicotine replacement therapies.

## 1. Introduction

Nicotine is the main substance that causes addiction to tobacco, although tobacco contains many toxic substances other than nicotine.^[1]^ In many countries, smoking cessation products or nicotine products, such as electronic (e-)cigarettes, nicotine sprays, nicotine gums, and nicotine patches, are sold as alternatives to tobacco.^[2–4]^ Nicotine products are more accessible than cigarettes. The liquid used in e-cigarettes is a mixture of high concentrations of nicotine, propylene glycol, and vegetable glycerin, and many cases of fatal intentional/unintentional poisoning have recently been reported.^[2,3,5–9]^ Nicotine patches stick to the skin and release nicotine for several days, and severe cases of poisoning from the use of patches have also been reported.^[10,11]^

Nicotine chewing gums are available in the market with nicotine contents of 2 mg and 4 mg, and according to the recommended dose of the American Centers for Disease Control and Prevention, depending on the amount of smoking, up to 24 can be consumed daily at 1-hour intervals.^[12]^ Compared with other nicotine alternatives, nicotine chewing gums are considered to be relatively safe due to the relatively small dose of nicotine; however, the risk of adverse effects from these gums is not negligible. This report presents the case of a patient who experienced severe adverse effects due to the consumption of a large number of nicotine chewing gums over several days.

## 2. Case presentation

A 29-year-old male patient experienced involuntary systemic tremors and convulsions; his mother called 119 when she saw him convulsing. He had a history of splenectomy due to a spleen injury caused by a traffic accident and was an ex-smoker. The patient had consumed 5 2-mg nicotine chewing gums twice daily, in the morning and evening, for 4 days (total amount is 70 mg). However, 3 hour before his visit, he consumed 15 gums at once and experienced continuous abdominal pain, nausea, vomiting, diarrhea, and headaches. Thirty minutes before visiting the emergency unit, he had collapsed due to limb weakness while going to the bathroom and convulsed; his mother called 119 for emergency treatment.

Upon arrival at the emergency unit, his consciousness was clear. Regarding vital signs, his blood pressure was 135/48 mm Hg, heart rate was 68 beats/minute, respiratory rate was 20 beats/minute, body temperature was 35.1 °C, and oxygen saturation was 98%. Arterial blood gas analysis showed that all investigated variables were within the normal range, except for arterial oxygen tension, which was 103 mm Hg; blood test results showed no specific findings except for an elevated white blood cell count of 14,800/µL, a prolactin level of 119.02 ng/mL (reference value: 3–25 ng/mL), and a lactic acid level of 6.4 mmol/L (reference value: 0.7–2.1 mmol/L); his chest radiographs showed no abnormal findings. Blood tests performed 2 hour later showed that his blood cotinine level was 3491 ng/mL (reference value: <25.0 ng/mL). Head computed tomography showed no specific findings.

Symptomatic treatment was started since acute nicotine poisoning was suspected, but the patient’s consciousness suddenly deteriorated 2 hour after admission, and he did not respond to pain stimuli. Pulse oximetry showed 54% saturation, and arterial blood gas analysis showed arterial carbon dioxide tension of 67.8 mm Hg, indicating acute respiratory failure. Therefore, tracheal intubation was performed, and mechanical ventilation was started. The patient was admitted to the intensive care unit, with continued mechanical ventilation. During hospital stay, his vital signs remained stable, and no additional involuntary tremors of the both limbs were observed. On the second hospital day, his consciousness became clear and his respiratory status improved; on the third day, the mechanical ventilator and tracheal tube were removed. Since then, he remained stable and was transferred to the general ward; he was discharged on the fifth hospital day without any specific sequelae.

## 3. Discussion

Tobacco, obtained from *nicotiana tabacum*, contains many toxic substances, including nicotine, which can be poisonous at high concentrations.^[1]^ Although nicotine is not carcinogenic, it can be toxic at high doses. Many alternatives to tobacco smoking, such as e-cigarettes, nicotine patches, nicotine candy, and nicotine chewing gums, are available.^[1]^ A nicotine patch is used to reduce the desire to smoke. The patch sticks to the skin, and the nicotine is absorbed continuously through the skin for several days.^[10]^ Nicotine candies and chewing gums are also used to reduce the desire to smoke. Chewing gums have a nicotine content of 2 mg or 4 mg and are meant to be chewed slowly for approximately 30 minute, one at a time; 24 gums can be consumed in a day at 1-hour intervals, depending on the smoking history.^[12]^ However, 39,402 cases of toxic exposure to tobacco/nicotine products in the United States have been reported over the past 5 years, of which 28 were of severe poisoning (less than 0.1%), which is extremely rare.^[4]^

Nicotine is absorbed into the body through the alveolar surface, oral mucosa, and intestinal mucosa; it then binds to the nicotine-acetylcholine receptors, affecting the autonomic nervous system, central nervous system, and motor nervous system.^[13]^ At low concentrations, nicotine acts as an antagonist in the distal part of the kidney, activating the secretion of neurotransmitters such as acetylcholine, dopamine, glutamate, norepinephrine, and serotonin; however, at high concentrations, it can cause inhibition of the acetylcholine receptor due to prolonged depolarization, resulting in bradycardia, hypotension, loss of consciousness, and respiratory failure.^[13]^ Treatment for nicotine poisoning includes symptomatic treatment, such as airway maintenance, circulatory support, and anticonvulsant therapy, if convulsions are present.^[1]^

The lethal dose of nicotine for adults is 30 to 60 mg (0.8–1 mg/kg); the half-life of nicotine is 2 to 3 hour.^[14,15]^ and the half-life of cotinine is 15 to 19 hour.^[15]^ For this reason, some lethal cases of poisoning from nicotine alternatives, blood nicotine levels of 190 to 13,600 ng/mL and blood cotinine levels of 1230 to 2500 ng/mL have been reported, indicating that cotinine has a narrow concentration range compared with nicotine.^[10]^ The nicotine from chewing gums is absorbed at a slower rate than that from tobacco or e-cigarettes, and the nicotine from the former remains in the bloodstream for several hours.^[16,17]^ In our patient, the nicotine level exceeded the toxic dose at 3491 ng/mL (reference value: <25.0 ng/mL), despite the fact that the single dose of each gum did not exceed the toxic level; however, he had consumed a large amount of gums at once after taking it for several days.

The limitations of this report are that the patient’s blood nicotine and cotinine levels were not measured at the follow-up. Nevertheless, it is a special case showing that the consumption of a large amount of chewing gums at once after using them for several days prior can increase the blood nicotine level to a toxic level and cause severe adverse effects such as loss of consciousness and respiratory failure.

Nicotine chewing gums are popular and can be purchased without a doctor’s prescription in many countries, including the Republic of Korea. Therefore, when using nicotine replacement therapies, such as nicotine chewing gums, it is crucial to explain the appropriate dosage and usage. In addition, one study.^[18]^ proposed the need to reevaluate the toxic dose because the currently used reference value for nicotine toxicity is based on a study published in 1906, and in that study, there was no significant difference in the blood nicotine levels of the survivors and dead patients. This case reported severe poisoning, despite the use of amounts that did not significantly exceed the known toxic dose; therefore, further studies on the toxicity of nicotine are needed, especially on the adverse effects of different products. Moreover, doctors who treat patients with suspected nicotine poisoning should carefully and continuously monitor them and provide appropriate treatment, such as airway management, respiratory support, and circulation maintenance, if the condition worsens.

## Acknowledgments

We would like to thank Editage (www.editage.co.kr) for English language editing.

## Author contributions

**Visualization:** Tae Chang Jang, Young Woo Seo.

**Writing – original draft:** Jung Eun Lee, Young Woo Seo.

**Writing – review & editing:** Jung Eun Lee, Young Woo Seo.
